# *Epimedium sagittatum* inhibits TLR4/MD-2 mediated NF-κB signaling pathway with anti-inflammatory activity

**DOI:** 10.1186/s12906-018-2363-x

**Published:** 2018-11-13

**Authors:** Ni Yan, Ding-Sheng Wen, Yue-Rui Zhao, Shun-Jun Xu

**Affiliations:** 1 Department of Research and Development, ImVin Pharmaceutical Co., Ltd, 2 Fangcaodian Road, Guangzhou, 510663 China; 2 School of Pharmaceutical Sciences, Sun Yat-Sen University, Guangzhou, 510006 China; 3 Zhuhai Jizhu Small and Medium Enterprises Advanced Technology Research Institute, Zhuhai College of Jilin University, Zhuhai, 519041 China

**Keywords:** *E, sagittatum*, Anti-inflammation, TLR4/MD-2, NF-κB

## Abstract

**Background:**

*Epimedium sagittatum* (Sieb.et Zucc.) Maxim., *Ying-Yang-Huo* in Chinese has been used as a traditional Chinese medicine and is deemed to “reinforce the kidney *Yang*”. Previous studies showed that *E*. *sagittatum* could modulate the immune system and treat some chronic disease such as rheumatic arthritis, cardiovascular diseases and osteoporosis. The aim of this study is to evaluate the anti-inflammatory effects of ethyl acetate extracts (YYHs) of *E*. *sagittatum* and its mechanisms of action.

**Methods:**

In order to explore the composition of YYHs, YYHs was analyzed using high performance liquid chromatography-mass spectrometry-mass spectrometry (HPLC-MS/MS) and in comparison with reference standards. Anti-inflammatory model was established in LPS-induced RAW264.7 cells. The levels of nitric oxide (NO) were measured with the Griess reagent. Production of tumor necrosis factor-alpha (TNF-α) and interleukin-2 (IL-2) were measured by enzyme-linked immunosorbent assays (ELISA). In addition, expression of p-p65 protein and TLR4/MD-2 complex was detected by western blots and flow cytometric, respectively. Nuclear factor kappa B (NF-κB) nuclear translocation was observed by fluorescence microscope.

**Results:**

A total of eight compounds were identified, of which icariside II was the most abundant compound. YYHs (12.5–50 μg/mL) had no obvious cytotoxic effect on cells, and remarkably inhibited LPS-induced production of NO, TNF-α and IL-2 with a dose-dependent manner. Additionally, YYHs up-regulated expression of p-p65 and TLR4/MD-2 complex. Further research showed that YYHs significantly suppressed NF-κB p65 nuclear translocation.

**Conclusion:**

In brief, YYHs contributed to the inhibition of LPS-induced inflammatory response through the TLR4/MD-2-mediated NF-κB pathway and may be a potential choice to combat inflammation diseases.

**Abstract graph:**

It includes a schema of pathways at the end of the paper.

## Background

Inflammation acts physiologically to protect normal host function after pathogen invasion or tissue damage [1]. As a major virulence factor of Gram-negative bacteria, LPS induces a serious systemic inflammatory response in macrophage [2]. TLR4 plays a key role for innate immune responses and its signaling is triggered by the transfer of its ligand LPS to a Toll-like receptor 4 /myeloid differentiation factor-2 (TLR4/MD-2) complex, which then undergoes homodimerization [3]. Thus, the TLR4/MD-2 complex constitute the indispensable components of the LPS receptor system and have been implicated in inflammation. Binding of the TLR4/MD-2 complex to LPS triggers the activation of JNK and NF-κB signaling pathway [4]. During inflammatory processes, LPS stimulates the production of the pro-inflammatory cytokines and inflammation mediators, such as TNF-α, IL-2 and nitric oxide (NO). However, inflammation persistently produces pro-inflammatory cytokines and inflammatory mediators that eventually evolves into excess inflammation, which is closely associated with many diseases including cancer [5], Alzheimer’s disease [6], obesity and diabetes [7]. In addition, extensive studies supported that anti-inflammatory therapy is efficacious to slow the progression and delay the onset of these disease.

Nuclear factor-κB (NF-κB) is a transcription factor that is essential for inflammation, immunity, cell proliferation and apoptosis [8]. There are two NF-κB signaling pathway including the classical and the alternative pathway, of which the classical attracts more attention. The classical pathway of NF-κB is triggered by pro-inflammatory cytokines and LPS. Subsequently, inhibitory protein IκB-α and p65 are phosphorylated, meanwhile, inhibitory molecule is degraded by proteasome-mediated. Then, the heterodimer p50/p65 is released and migrats from the cytoplasm to the nucleus to regulate the expression of multiple target genes, such as TNF-α, IL-6, iNOS and so on [9, 10].

*Epimedium sagittatum* (Sieb.et Zucc.) Maxim., a tranditional herbal drug in China, widely grows in Guangdong, Guangxi and Jiangxi province. And it contains flavonoids, lignans and so forth. Specially, major secondary metabolites of *E*. *sagittatum*, in which mainly includes epimedin A, B, C and icariin [11]. Species of Epimedium have four members according to the Chinese Pharmacopoeia, they are *E. brevicornu* Maxim., *E. sagittatum* (Sieb.et Zucc.) Maxim., *E. pubescens* Maxim. and *E. koreanum* Nakai [12]. Previous studies showed that the species of Epimedium exhibited many bioactivities, such as antiviral [13], antinociceptive [14], anti-aging [15], antioxidant, neurotective effect [16], enhancing immunity [17] and promoting estrogen biosynthesis [18]. However, there are few reports about the biological activity of *E. sagittatum*, especially in terms of anti-inflammatory.

The objective of this study is to analyze constituent and evaluate the anti-inflammatory activity of YYHs by LPS stimulated RAW 264.7 cells model. Besides, we tried to explore the anti-inflammation mechanism via the TLR4/MD-2-mediated NF-κB signaling pathway.

## Methods

### Preparation of extract

The dried material was from Guangdong province and provided by ImVin Pharmaceutical Co., Ltd. (Guangzhou, China). The authentication of plant materials was finished by professor Dingping (Guangzhou University of Chinese Medicine). At first, the herb of YYH was powdered and heat extracted three times with 10-fold of 50% ethanol at 70 °C for 25 min. After cooling, the solution was filtered and removed ethanol, followed by separations with ethyl acetate (*v*/v = 1:1). The YYH extracted ethyl acetate fraction was concentrated by rotary evaporation (EYELA, Japan) and stored at − 20 °C before use.

### Chemicals and reagents

3-(4,5-dimethylthiazol-2-yl)-2,5-diphenyltetrazoliumbromide (MTT), Griess reagent and bacterial lipopolysaccharide (LPS) were acquired from Sigma Chemical Co. (St Louis, MO, USA). Fetal bovine serum (FBS) was purchased from Life Technologies (Auckland, New Zealand). Dulbecco’s Modified Eagle’s Medium (DMEM), Penicillin-streptomycin solution, Dulbecco’s Phosphate Buffered Saline (DPBS), GlutaMAX™-1 and 0.25% Trypsin-EDTA were obtained from Hyclone (Logan, Utah, USA). PE-conjugated rat anti-mouse TLR4/MD-2 complex (clone MTS510) and rat IgG2a kappa isotype control antibodys were products of eBioscience (San Diego, CA, USA). Mouse TNF-α and IL-2 ELISA kits were acquried from Neobioscience Technology Co., Ltd. (Beijing, China). NF-κB Activation –Nuclear Translocation Assay kit from Beyotime Institute of Biotechnology (Nanjing, China). Rabbit phospho-NF-κB (Ser536) antibody and rabbit NF-κB p65 (C22B4) antibody from Cell Signaling Technology (Danvers, MA) were also obtained. Epimedin C and icariin were provided by the National Institute for Food and Drug Control (Beijing, China). Epimedin B was purchased from Herbest (Baoji, China). Baohuoside II and Baohuoside VII were obtained from Chem Faces (Wuhan, China). All standards had a purity of 98%.

### HPLC and LC-MS/MS conditions

Chromatographic separation (Agilent 1100 HPLC system) was achieved on an Agilent Zorbax SB-C18 column (250 × 4.6 mm, 5 μm). The mobile phase consisted of acetonitrile as solvent A, methanol as solvent B and 0.5% acetic acid in water as solvent C. The gradient of mobile phase was shown in Table 1. The flow rate was 1 mL/min, and the column temperature was set at 20 °C. The diode-array detection was set to monitor at 270 nm. The mass spectrometry analysis was performed on a LTQ-Orbitrap XL mass spectrometer (Thermo Electron, Bremen, Germany) coupled with an ESI source, and used with the following conditions: source temperature: 350 °C; ion spray voltage - 4.5 kV; Gas 1, Gas 2,curtain gas, and collision gas (nitrogen) were separately set at 50, 50, 45, and 12 psi; sheath gas flow rate, 40 arbitrary units; auxiliary gas flow rate, 5 arbitrary units; electrospray voltage, 3.5 kV; capillary voltage, -32 V; capillary temperature, 270 °C.

**Table 1:** Mobile phase condition of chromatographic separation

Time (min)	Acetonitrile (%)	Methanol (%)	0.5% acetic acid in water (%)
0–30	12–25	0	88–75
30–45	25–23.5	0–11	75–65.5
45–68	23.5–35	11–4	65.5–61
68–85	35	4	61
85–90	35–50	4–0	61–50
90–95	100	0	0
95–110	100	0	0

### Cell culture and viability assay

RAW 264.7 cells were obtained from American Type Culture Collection (Manassas, VA, USA) and cultured in DMEM which was supplemented with 10% FBS, 1% penicillin-streptomycin solution and 1% GlutaMAX™-1 at 37 °C in a humidified incubator containing 5% CO_2_. Cells were seeded in 96-well plates at a density of 1 × 10^5^ cells/mL and incubated for 24 h. They treated with different concentrations of YYHs (12.5, 25, 50, 75 μg/mL) in the absence or presence of 1 μg/mL LPS for 24 h, respectively. Then, 50 μL MTT solution (0.5 mg/mL in DPBS) was added to plates and incubated for 4 h in the incubator. After incubation finished, the supernatants was discarded and replaced with DMSO (200 μL) to dissolve the formazan crystal. The absorbance of each well was detected at 570 nm using a microplate reader (BERTHOLD Technologies, Germany).

### Nitric oxide (NO) and enzyme-linked immunosorbent assay

To assay the production of NO, IL-2 and TNF-α, the supernatant of RAW 264.7 cells was collected after co-treated with YYHs (12.5, 25, 50 μg/mL) and LPS (1 μg/mL) for 24 h. The IL-2 and TNF-α were determined using enzyme-linked immunosorbent assay kits according to the manufacturer’s instructions. Meanwhile, the NO production was determined by mixing 100 μL of the supernatant with an equal volume of Griess reagent comprising 50 μL of 2% sulfanilamide in 4% phosphoric acid and 50 μL of 0.2% N-(1-naphthyl) -ethylenediamine dihydrochloride in water for 10 min at room temperature, and the concentration of nitrite was determined by using a standard curve generated with sodium nitrite under a spectrophotometer on a wavelength of 550 nm.

### Flow cytometric analysis

RAW 264.7 cells treated with YYH extracts (12.5, 25 and 50 μg/mL) and LPS (1 μg/mL) for 18 h were harvested with 0.25% trypsin and washed with PBS by centrifugation for 5 min. Subsequently, cells were incubated with a PE-conjugated anti-mouse TLR4/MD-2 complex antibody and FITC-conjucted anti-mouse CD14 or PE/FITC-conjugated IgG as isotype control for 30 min at 4 °C, respectively. The cell surface makers expression was analyzed by Attune acoustic focusing cytometer (Thermo Fisher Scientific, USA). The data of MFI value were calculated the levels of fluorescence by Flow Jo software (FlowJo LLC, USA).

### Nuclear factor-κB (NF-κB) nuclear translocation assay

The activation of NF-κB nuclear translocation was detected by using NF-κB activation nuclear translocation assay kit according to the manufacturer’s protocol. Briefly, cells were pretreated YYHs (50 μg/mL) for 1 h prior to incubation with LPS for another 1 h, together. After fixing and permeabilizing, the cells were incubated with a blocking solution for 1 h, followed by NF-κB p65 antibody at 4 °C overnight. Next, after washing three times, cells incubated with a Cy3-conjugated secondary antibody for 1 h, then with DAPI for 5 min before observation. The activation of NF-κB p65 was visualized with a fluorescence microscope (Axiovert 40 CFL, Carl Zeiss) at excitation wavelength of 350 nm for DAPI and 540 nm for Cy3, and the red and blue images were overlaid by Image-pro plus 5.1 software to indicated the areas of co-localization.

### Western blot assay

RAW 264.7 cells (7.5 × 10^5^ cells/mL) were seeded in 6-well plates for 24 h. Then, cells orderly incubated with YYHs (2 h) and LPS (1 h). The total proteins, extracted with cell lysis buffer (RIPA:PMSF: Phosphatase inhibitor = 100:1:1) by ultrasonic, were quantified using a BCA protein assay kit. An equal amount of protein (25μg) was separated on 10% SDS-PAGE for electrophoresis, followed by transfer to PVDF membranes. Then, the membranes were severally blocked with 5% non-fat milk or 5% BSA for 1 h and then incubated with primary antibodies (1:1000 dilution) overnight at 4 °C. The following day, membranes were incubated with a horseradish peroxidase-conjugated goat anti-rabbit antibody (1:1000 dilution) for 2 h and detected by enhanced chemiluminescence (ECL). Protein levels were normalized against included β-actin standards and analyzed by Image J software.

### Statistical analysis

All experiments were performed at least three times and expressed as mean ± standard deviation (S.D). The differences between groups were analyzed by one-way ANOVA, followed by a Tukey’s test using SPSS 20.0 software (SPSS Inc., Chicago, USA). *P* value of 0.05 or less was considered as statistically significant.

## Results

### Measurement of major compounds of YYHs

**Fig. 1:**
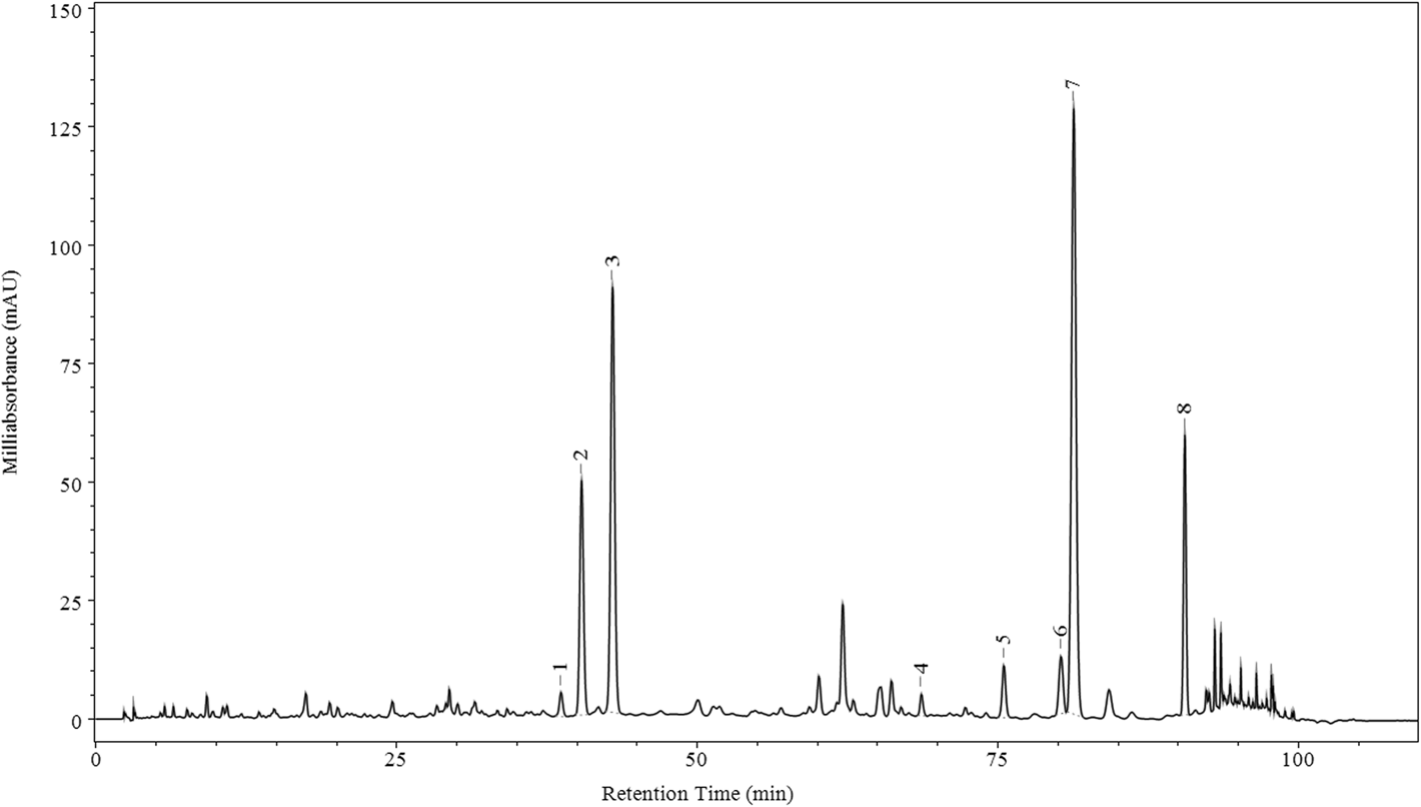
Representative HPLC chromatogram of YYHs at 270 nm

A total of eight chromatographic peaks were identified five of which were assigned by comparison with reference standards, three of which were characterized based on their chromatographic behavior and MS/MS fragmentation pattern. As shown in Fig. 1 and Table 2, sagittatoside B ([M-H]^−^ *m/z* at 645.21472; error ppm = 4.746), 2″-O-rhamnosyl icariside II ([M-H]^−^ *m/z* at 659.23071; error ppm = 4.129) and icariside II ([M-H]^−^ *m/z* at 513.17407; error ppm = 2.832) were detected by LC-MS/MS. Epimedoside C, icariin, icariside II and 2″-O-rhamnosyl Icariside II were the major compounds of YYHs, which were 9.59, 18.89, 30.94 and 7.97%, respectively.

**Table 2:** The main components of YYHs

Peak	Retention time (min)	Component	Concentration (%)
1^a^	38.664	Epimedoside B	0.90
2^a^	40.376	Epimedoside C	9.59
3^a^	42.963	Icariin	18.89
4^a^	68.647	Baohuoside II	0.70
5^a^	75.499	Baohuoside VII	2.07
6	80.248	Sagittatoside B	2.94
7	81.308	2″-O-Rhamnosy (Icariside II)	30.94
8	90.552	Icariside II	7.97

(1–5) ^a^were assigned by comparison with reference standards. (6–8) were identified by MS/MS

### Cytotoxicity of YYHs on viability of cells

**Fig. 2:**
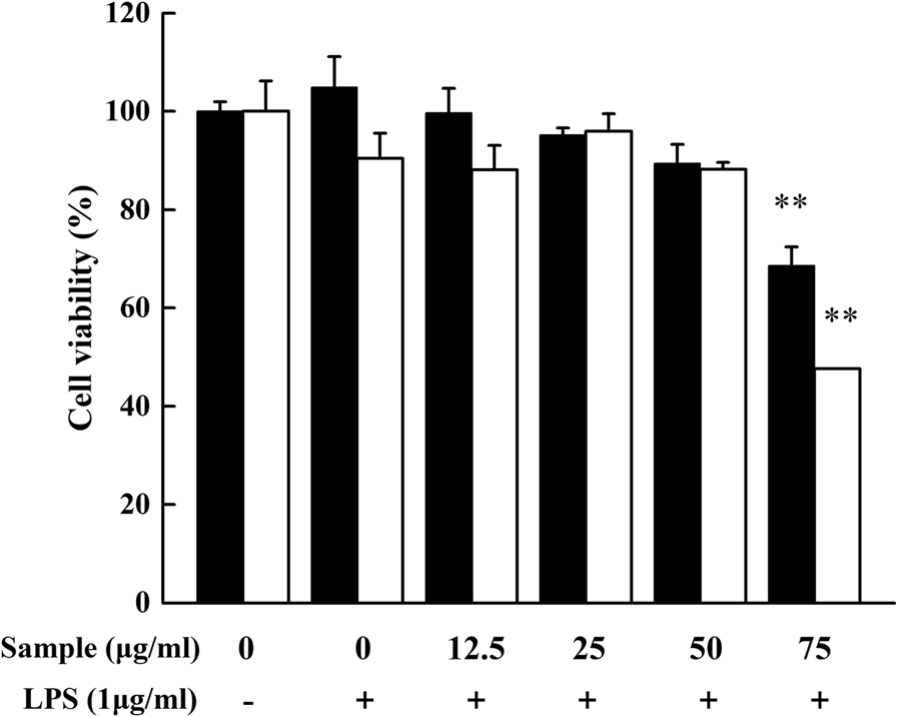
Cytotoxicity of YYHs on RAW 264.7 cells. Cells were treated in the presence of YYHs or in combination with LPS (1 μg/mL) for 24 h. Cell viability was determined by MTT assay (black bar, YYHs treated; white bar, YYHs+LPS treated). Compared with the control, ^#^*P* < 0.05, ^##^*P* < 0.01. Compared with LPS group, ^*^P < 0.05, ^**^P < 0.01

The MTT assay was carried out to estimate the cytotoxicity of YYHs on RAW 264.7 cells. As the results shown in Fig. 2, the presence of LPS (1 μg/mL) had no impact on cell viability, expectedly. Then, exposure of cells to (12.5–50 μg/mL) YYHs with 1 μg/mL LPS for 24 h showed no cytotoxicity. However, when the cells were treated with 75 μg/mL YYHs and 1 μg/mL LPS, the cell viability was less than 50%. Therefore, all subsequent experiments were performed at nontoxic concentrations (12.5–50 μg/mL).

### Effect of YYHs on levels of NO, TNF-α and IL-2

**Fig. 3:**
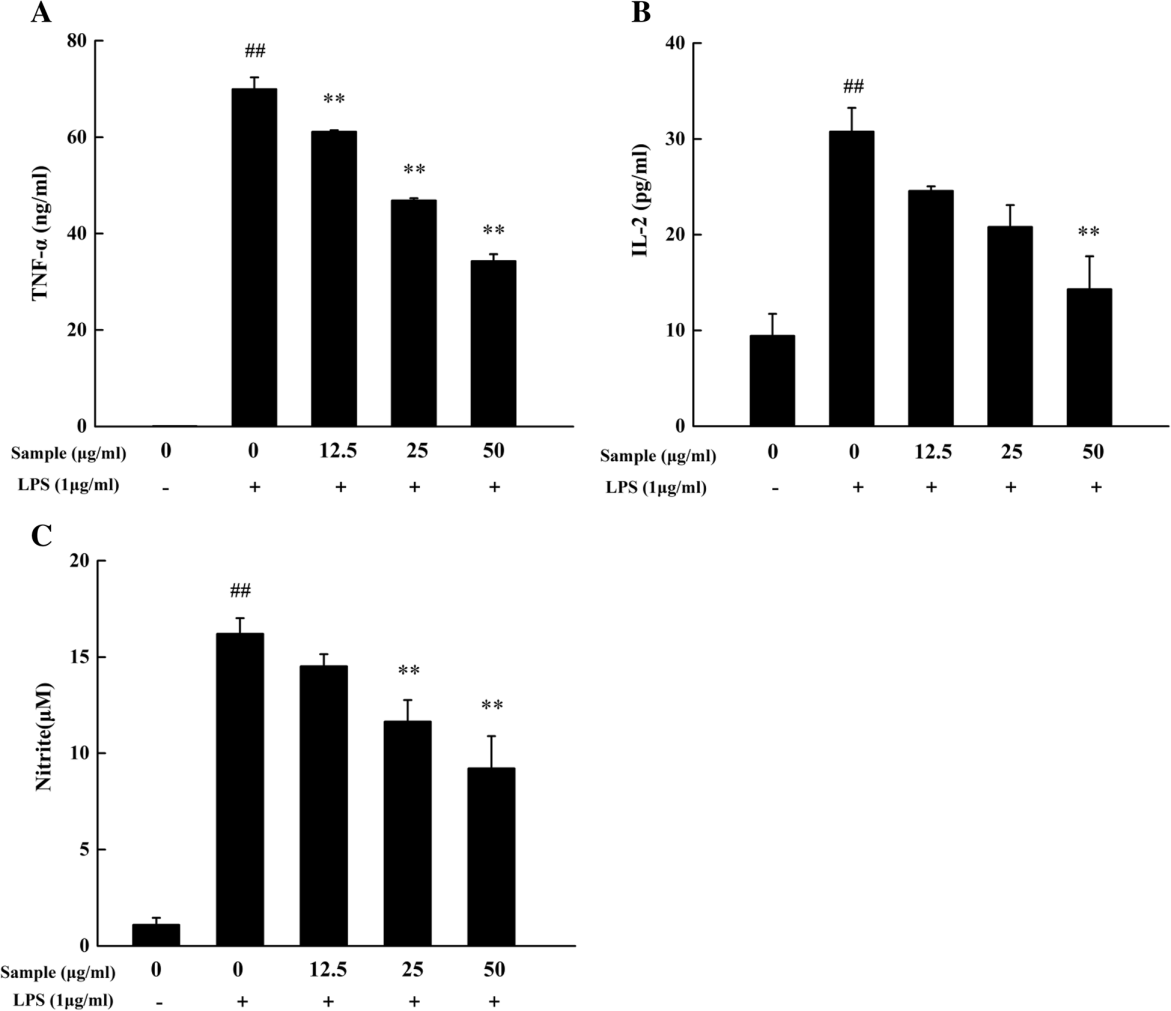
Effect of YYHs on TNF-α (**a**), IL-2 (**b**) and NO (**c**) production in LPS-induced RAW 264.7 cells. Cells were pretreated with YYHs (12.5–50 μg/mL) and LPS for 24 h and collected culture supernatant. The culture supernatant was detected to ELISA kits and Griess reagent, respectively. Compared with the control, ^#^*P* < 0.05, ^##^*P* < 0.01. Compared with LPS group, ^*^P < 0.05, ^**^P < 0.01

We first investigated the anti-inflammatory activity of YYHs. Treatment of LPS for 24 h can lead to inflammatory response on RAW 264.7. During inflammation, a large amount of NO and pro-inflammatory cytokines including TNF-α and IL-2 were generated. YYHs at dose of 50 μg/mL significantly decreased the levels of TNF-α and IL-2 with inhibition values of 50.89 ± 3.55% and 55.38 ± 7.60% (Fig. 3A, B). Furthermore, YYHs concentration-dependently suppressed the production of NO (Fig. 3C).

### Effect of YYHs on cell surface of TLR4/MD-2 complex in cells

**Fig. 4:**
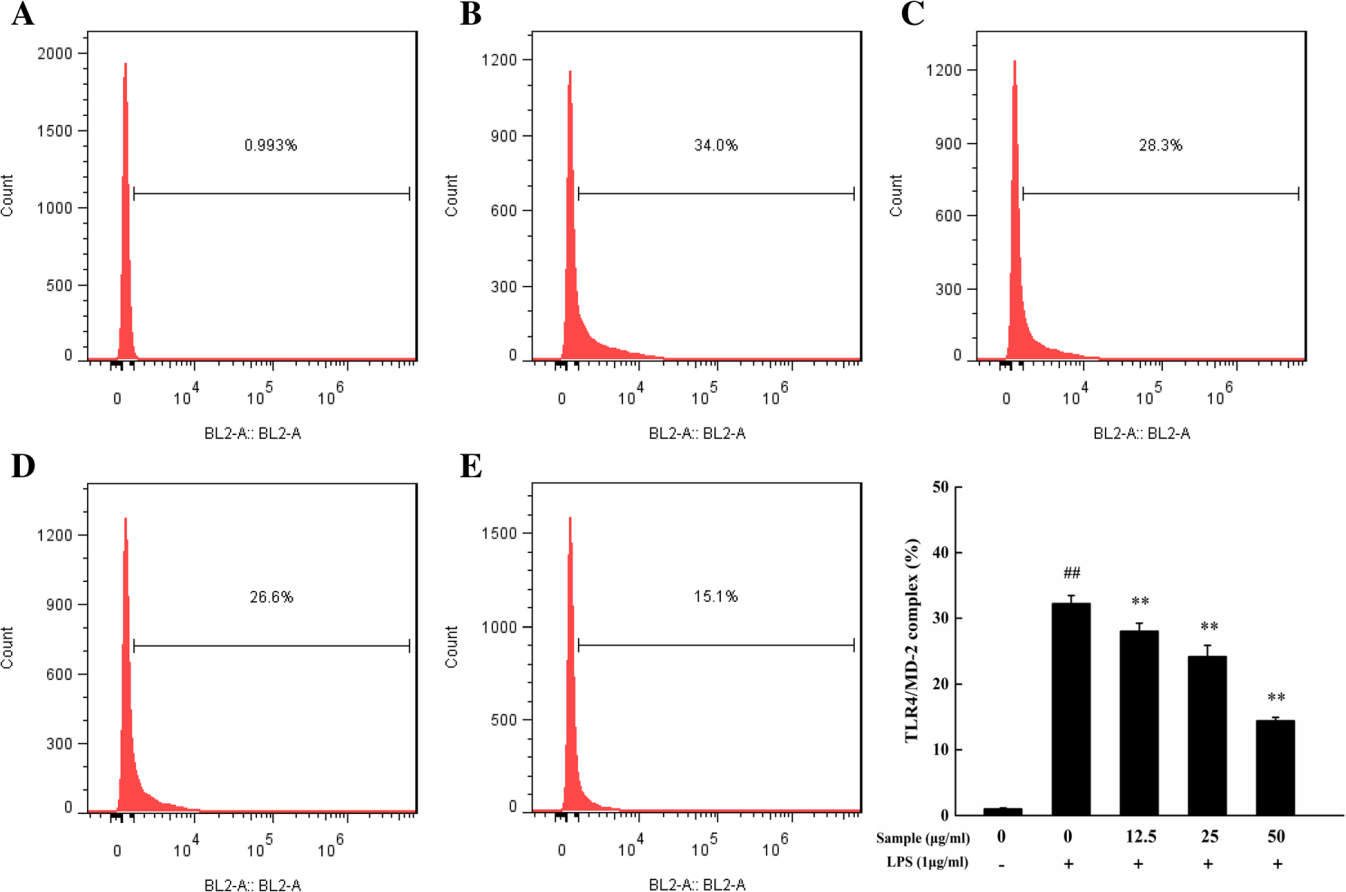
Flow cytometric analysis for TLR4/MD-2 complex. Cells were treated with YYHs and LPS (1 μg/mL) for 18 h. And TLT4/MD-2 complex was detected with FCM by PE conjugated mAb TLR4/MD-2 complex antibody (**a**, blank group; **b**, LPS group; **c**-**e**, 12.5–50 μg/mL YYHs group). Compared with the control, ^#^*P* < 0.05, ^##^*P* < 0.01. Compared with LPS group, ^*^P < 0.05, ^**^P < 0.01

The TLR4/MD-2 complex was critical for LPS recognition and innate immune response. To assess the effect of YYHs on the formation of TLR4/MD-2 complex, we detected the expression of the complex by flow cytometry. As shown in Fig. 4, The plenty of TLR4/MD-2 complex were formed after LPS stimulated cells, and treatment of YYHs can interrupt the formation of the TLR4/MD-2 complex in a concentration-dependent manner. When the concentration of 50 μg/mL, TLR4/MD-2 complex obviously decreased more than half as LPS group.

### Effect of YYHs on the nuclear translocation and phosphorylation of p65 in cells

**Fig. 5:**
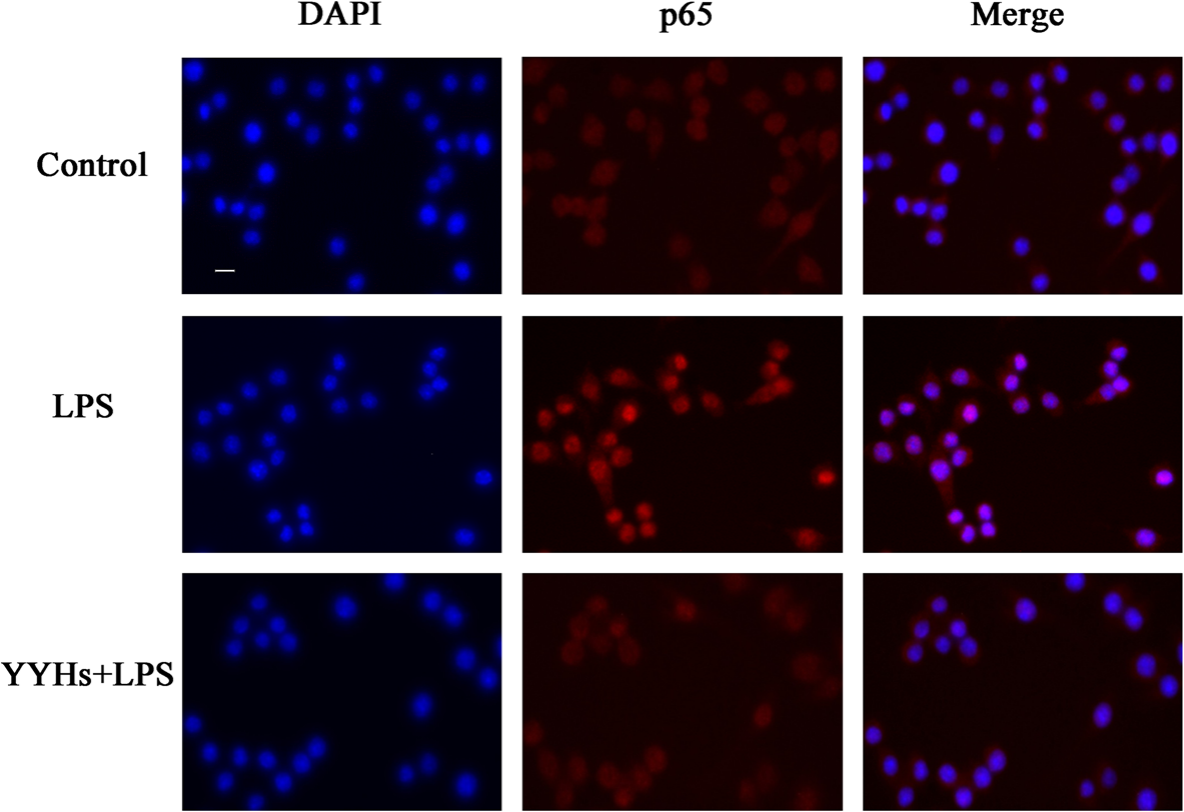
Effect of YYHs on NF-κB activation in RAW 264.7 cells. The cells were seeded in a 6-well culture plate, and treated with YYHs for 1 h and followed by stimulated with LPS for another 1 h. After treatment, fixed cells were incubated with p65 antibody and Cy3-conjugated secondary antibody, and nuclei were stained with DAPI. The images were obtained by fluorescence microscopy and overlay (Control, untreated cells; LPS, LPS (1 μg/ml) only; YYHs+LPS, YYHs (50 μg/mL) + LPS)

**Fig. 6:**
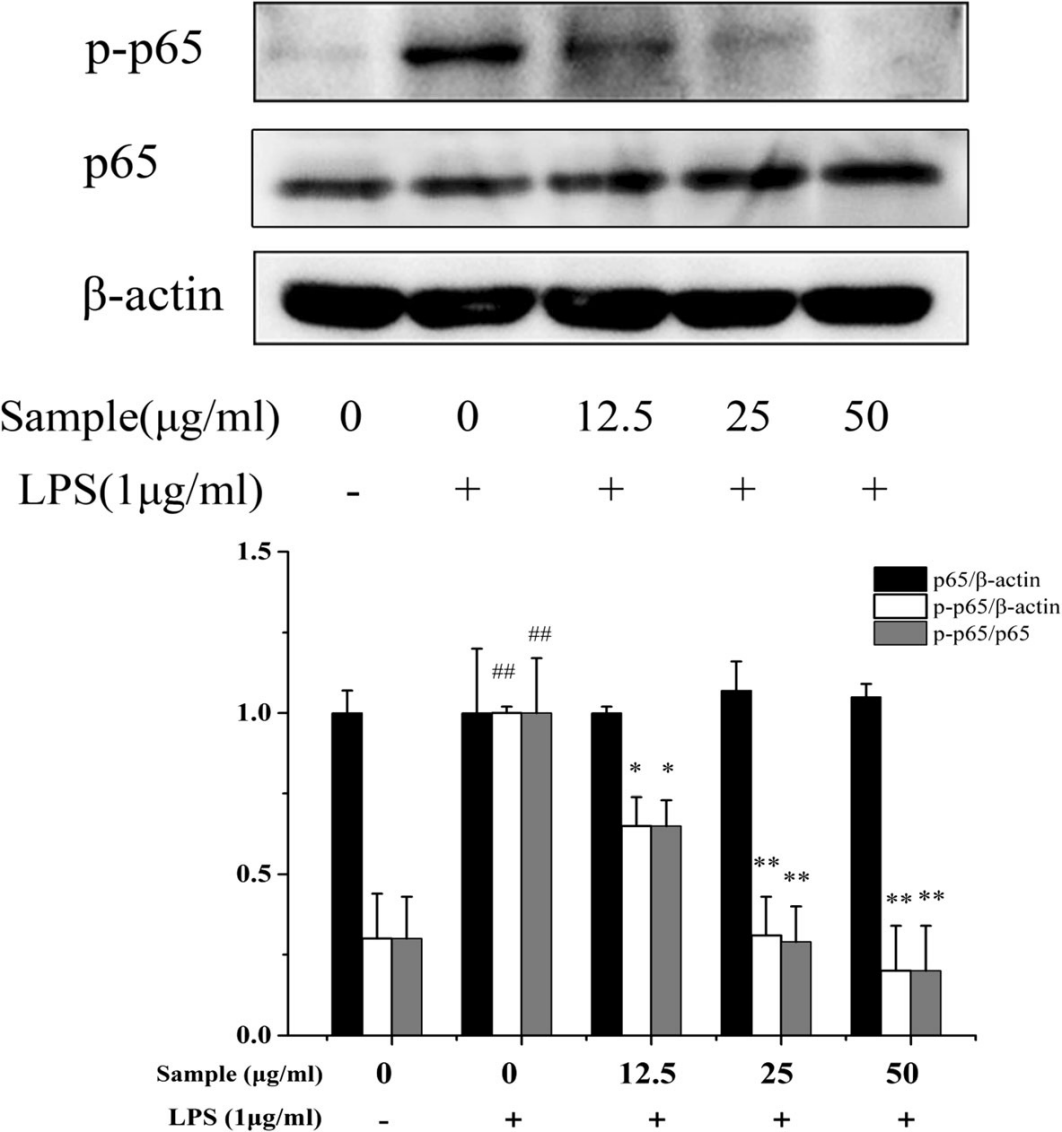
Effect of YYHs on LPS-induced activation of NF-κB pathway in RAW 264.7 cells. The cells were pretreated with different concentrations of YYHs for 2 h and then stimulated with LPS(1 μg/mL) for another 1 h. The NF-κB p65 and phosphorylated p65 in the total protein was detected by western blotting. β–acting was used as the internal control for normalization. Compared with the control, ^#^*P* < 0.05, ^##^*P* < 0.01. Compared with LPS group, ^*^P < 0.05, ^**^P < 0.01

As an important upstream transcription factor, NF-κB regulated expression of NO and .pro-inflammatory cytokines by LPS stimulation. Translocation of NF-κB p65 into the nucleus were increased within 1 h after LPS stimulation, as compared with control groups (Fig. 5). However, pre-treatment with YYHs (50 μg/mL) for 1 h markedly suppressed NF-κB p65 levels in the nucleus. Meanwhile, we explored the effect of YYHs on LPS induced NF-κB p65 phosphorylation by western blot analysis. Compared to the unstimulated cells, LPS expectedly induced phosphorylation of p65. Treatment with different concentration of YYHs attenuated phosphorylation of p65 in a dose-dependent manner (Fig. 6). These results strongly suggested that YYHs down-regulated the LPS–induced phosphorylation of p65.

## Discussion

Traditionally, the leaves of *E*pimedium had long been used as a kidney tonic and antirheumatic for more than 2000 years [19]. In recent years, it was also used to treat various diseases, such as parkinson’s disease [20], rheumatic arthritis [21], osteoporosis [22, 23] and asthma [24]. Virtually, most of the attention focused on *E*. *brevicornu* Maxim and *E*. *koreanum* Nakai. In this study, Enzyme-linked immunosorbent assay, flow cytometric analysis and western blot assay which were performed under different YYHs exposure time was according to the references described as previous [4, 25]. However, it was found that a small proportion of the cells in the 6-well plate were shed 24 h post stimulated with LPS under the premise of the same cell plating density, when shortening the stimulation time of co-treatment to 18 h, there was no sheding of cells. So we the exposure different time was employed in assays. Finally, the results showed YYHs distinctly decreased production of NO, TNF-α and IL-2, and markedly suppressed the expression of TLR4/MD-2 complex and NF-κB p65 nuclear translocation, which implied YYHs had anti-inflammatory activity on LPS-induced inflammatory responses.

In our study, YYHs were detected by HPLC system. Among the components, 2″-O-rhamnosyl icariside II was the richest component (30.94%) in ethyl acetate fraction. As a prenylated flavonol glycosides, 2″-O-rhamnosyl icariside II was isolated from Epimedium koreanum Nakai for the first time in 1991 [26]. Additionally, icariin (18.89%), epimedoside C (9.59%) and icariside II (7.97%) are relatively abundant in the extracts, and it is known to all that those were some major bioactive components in Herba Epimedii. Icariin exerted its anti-inflammatory effect mainly through modulating glucocorticoid receptor function and inhibiting the pro-inflammatory transcription factors including TNF-α, IL-6, IL-8 and MMP-9 [27, 28]. Previous studies demonstrated that icariin protected dopamine neurons against neurotoxins-induced neurotoxicity and inhibited pro-inflammatory factors production via the NF-κB signaling pathway, in which might be compactly associated with the inhibition of microglia-mediated neuroinflammation [29]. Furthermore, Icariside II also possibly suppressed neuroinflammation by reducing expression of IL-1, IL-1β, TNF-α, COX-2, and iNOS mRNA and protein, which eventually reversed Aβ-induced cognitive deficits [30]. Through rat model of experimental autoimmune encephalomyelitis (EAE), researchers found that epimedium flavonois (icaiin accounting for 56.7% and epimedin C 20.6%) can alleviate demyelination and inflammatory infiltration [31].

NO is a relatively short –lived free radical, which participates in physiological processes and is synthesized by nitric oxide sythase (NOS) and L-Arginine. NOS divides into three isoforms, such as inducible NOS (iNOS), endothelial NOS (eNOS) and neuronal NOS (nNOS), of which iNOS mainly regulate inflammation responses [25, 32]. It is well-know that inflammation response by secreting inflammatory mediator and pro-inflammatory cytokines could be caused by the stimulation of LPS. Overproduction of NO and TNF-α may lead to DNA damage by oxidative stress and DNA mutation [33–36]. In addition, there are several evidences that IL-2 has an association with chronic inflammation by modulating production of the pro-inflammatory cytokine (IFN-γ) [37]. In our study, we found that treatment of YYHs effectively modulated NO production and attenuates the expression of TNF-α and IL-2 in active macrophages (Fig. 3). And it means YYHs may be a valuable anti-inflammatory agent.

According to the crystal structure research, multiple structural components of TLR4/MD-2 are involved in LPS recognition [38]. TLR4/MD-2 complex as the main LPS binding receptors, it responds to inflammatory stimuli as well as mediate NF-κB signaling pathway in macrophages. For example, Zhankuic acid A contributes to the regulation of inflammatory responds through TLR4/MD-2 mediating MAPK and NF-κB pathway, which the same applies to procyanidin B1 and Baicalein [39–41]. NF-κB is critical downstream target of TLR4/MD-2 pathway and adjusts the expression of pro-inflammatory and inflammatory mediator genes [42]. Previous studies revealed that apigenin was attributed to decreasing production of IL-6, IL-1β and TNF-α through the inhibition of NF-κB activation [43]. Besides, indirubin also effectively suppressed LPS-induced inflammation through the NF-κB pathway [44]. Similarly, we demonstrated that YYHs actively ameliorated the inflammatory responses induced by LPS. Finally, YYHs inhibited the formation of TLR4/MD-2 complex (Fig. 4), which subsequently down-regulated the phophorylation of p65 (Fig. 6) and restrained translocation of p65 into the nucleus (Fig. 5).

## Conclusions

In summary, YYHs from *E.sagittatum* remarkedly decreased the production of inflammatory mediator and pro-inflammatory cytokines in LPS-induced RAW264.7 cells, and that interacted with TLR4/MD-2 complex formation to block LPS action. Furthermore, YYHs inhibited the phosphorylation of p65 in total cell lysates and the translocation of p65 from the cytoplasm to the nucleus. In general, YYHs contributed to ameliorate inflammatory response through TLR4/MD-2 mediating NF-κB pathway. This study provides novel insights into the mechanisms of YYHs as anti-inflammatory agents to LPS-mediated inflammatory response. Moreover, further studies needs to be verified in animal models.

## Abbreviations

HPLC-MS/MS: High performance liquid chromatography-mass spectrometry-mass spectrometry
IL-2: Interleukin-2
LPS: Lipopolysaccharide
NF-κB: Nuclear factor-κB
NO: Nitric oxide
TLR4/MD-2: Toll-like receptor 4/ myeloid differentiation factor-2
TNF-α: Tumor necrosis factor-α
YYHs: *E.sagittatum* of ethyl acetate extracts
